# Cystatin C protects neuronal cells against mutant copper-zinc superoxide dismutase-mediated toxicity

**DOI:** 10.1038/cddis.2014.459

**Published:** 2014-10-30

**Authors:** S Watanabe, T Hayakawa, K Wakasugi, K Yamanaka

**Affiliations:** 1 Department of Neuroscience and Pathobiology, Research Institute of Environmental Medicine, Nagoya University, Nagoya, Aichi, Japan; 2 Laboratory for Motor Neuron Disease, RIKEN Brain Science Institute, Wako, Saitama, Japan; 3 Department of Life Sciences, Graduate School of Arts and Sciences, The University of Tokyo, Komaba, Meguro-ku, Tokyo, Japan

## Abstract

Amyotrophic lateral sclerosis (ALS) is a fatal neurodegenerative disease characterized by the selective and progressive loss of motor neurons. Cystatin C (CysC), an endogenous cysteine protease inhibitor, is a major protein component of Bunina bodies observed in the spinal motor neurons of sporadic ALS and is decreased in the cerebrospinal fluid of ALS patients. Despite prominent deposition of CysC in ALS, the roles of CysC in the central nervous system remain unknown. Here, we identified the neuroprotective activity of CysC against ALS-linked mutant Cu/Zn-superoxide dismutase (SOD1)-mediated toxicity. We found that exogenously added CysC protected neuronal cells including primary cultured motor neurons. Moreover, the neuroprotective property of CysC was dependent on the coordinated activation of two distinct pathways: autophagy induction through AMPK-mTOR pathway and inhibition of cathepsin B. Furthermore, exogenously added CysC was transduced into the cells and aggregated in the cytosol under oxidative stress conditions, implying a relationship between the neuroprotective activity of CysC and Bunina body formation. These data suggest CysC is an endogenous neuroprotective agent and targeting CysC in motor neurons may provide a novel therapeutic strategy for ALS.

Failure of protein quality control and degradation is deeply involved in the pathomechanisms of neurodegenerative diseases. Prominent deposition of disease-specific proteins is characteristic in neurodegenerative diseases, such as amyloid-*β* in Alzheimer's disease or huntingtin in Huntington's disease. Amyotrophic lateral sclerosis (ALS) is a fatal adult-onset neurodegenerative disease characterized by the selective loss of motor neurons. While 90% of ALS is sporadic, 10% is inherited. Among the inherited ALS cases, dominant mutations in Cu/Zn superoxide dismutase (SOD1) are the frequent cause of inherited ALS.^1^ Transgenic mice and rats expressing a human gene for SOD1 with an ALS-linked mutation develop an ALS phenotype, whereas those with deletion of wild-type SOD1 do not, indicating that acquired toxicity mediated by mutant SOD1 is involved in neurodegeneration.^2,3^ In SOD1-linked ALS, SOD1-containing inclusions or oligomerized protein complexes have been specifically found in the spinal motor neurons and astrocytes.^4^ It has been proposed that mutant SOD1 proteins are misfolded and consequently aggregated, gaining toxic properties at some stage in their formation.^5^ Furthermore, recent studies have suggested that the accumulation of misfolded SOD1 proteins is involved in the pathomechanisms of sporadic ALS.^6,7^ Therefore, a reduction of misfolded SOD1 proteins might be one of the viable therapeutic approaches for ALS.

Cystatin C (CysC) is an endogenous cysteine protease inhibitor and expressed in various tissues.^8^ In the central nervous system, CysC is mainly secreted from the choroid plexus into the cerebrospinal fluid. CysC is a member of the type-II Cystatin family and inhibits cathepsin B, S and F.^9^ Although its precise function, especially in the central nervous system, is still uncertain, some studies have revealed that CysC has a neuroprotective role in neurodegenerative diseases.^10^ In a mouse model for Alzheimer's disease, overexpression of human CysC in the mice reduced deposits of amyloid-*β* fibrils.^11^ CysC has been shown to improve the survival of dopaminergic neurons in a rat model of Parkinson's disease.^12^ In sporadic ALS, CysC is a major component of Bunina bodies, which are ALS-specific inclusion bodies, found in remaining motor neurons,^13^ and the levels of CysC are decreased in the cerebrospinal fluid of ALS patients.^14,15^ Intriguingly, it was also reported that the concentration of CysC in the cerebrospinal fluid is correlated with the survival time of ALS patients,^15^ implying a potent neuroprotective property of CysC in ALS.

Previous reports showed that CysC induces autophagy to protect neuronal cells against various stresses including serum or growth-factor deprivation and oxidative stresses.^10,16^ Autophagy is a major intracellular proteolytic pathway that targets misfolded or aggregated proteins as well as the ubiquitin-proteasome pathway. Because the ubiquitin-proteasome pathway is impaired in both SOD1-linked^17,18^ and SOD1-unrelated^19,20^ ALS models, autophagy activation may complementally degrade the abnormal proteins to rescue motor neurons. Indeed, involvement of autophagy is implicated in the experimental models of ALS.^21,22^ Moreover, recent studies have shown that cathepsin B (CatB), a member of the cysteine protease family that is inhibited by CysC, is deeply involved in motor neuronal degeneration. Increased immunoreactivity of CatB was often found in the neurons of sporadic ALS patients^23^ or ALS model mice^24^ and CatB-knockout mice showed a lower rate of motor neuron death after nerve injury,^25^ suggesting that inhibition of CatB is beneficial for motor neuronal survival. These previous data suggest the possibility that CysC is a promising therapeutic candidate for ALS. However, no evidence has been provided for the role of CysC in neuroprotection in ALS models.

Here, we performed direct tests of the neuroprotective property of CysC using neuroblastoma cell Neuro2a (N2a) and primary mix-cultured motor neurons derived from mutant SOD1 transgenic mice and identified that CysC is a novel neuroprotective agent against mutant SOD1-mediated neurotoxicity that acts through induction of autophagy and inhibition of CatB.

## Results

### CysC is enriched in the healthy motor neurons in mouse spinal cords

**Figure 1:**
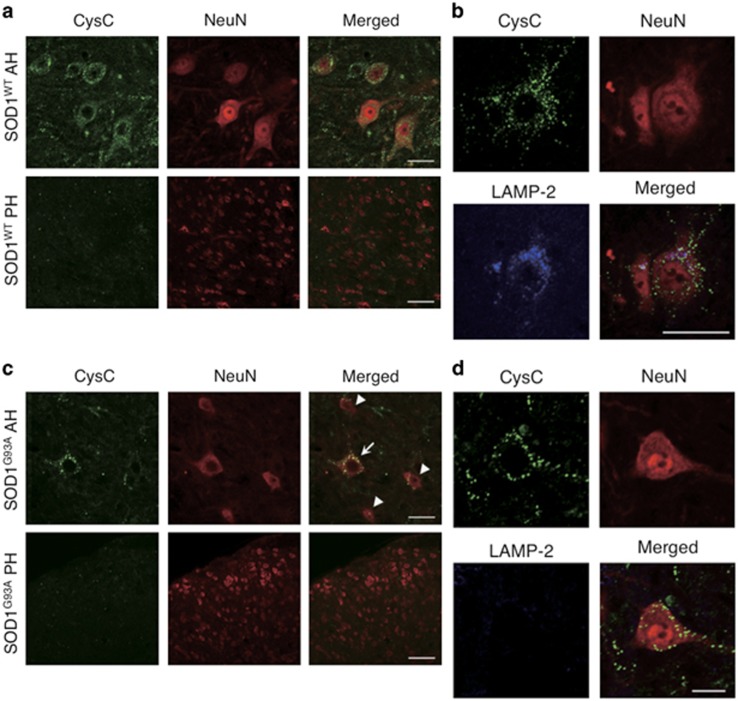
CysC expression in wild-type and mutant SOD1 mouse spinal cords. Double or triple immunostaining for neurons, lysosomes and Cystatin C was performed using antibodies for NeuN, LAMP-2 and CysC, respectively. Transverse sections in the anterior horn (AH) and the posterior horn (PH) of 5-month-old SOD1^WT^ (**a** and **b**) or SOD1^G93A^ (**c** and **d**) mouse spinal cords were analyzed by confocal microscopy. The arrow represents the remaining normal-shaped neuron, and the arrowheads represent the shrunken neurons, respectively. Scale bars: 50 *μ*m in **a**–**c**, 25 *μ*m in **d**

Although Bunina bodies are the specific structures in patients with sporadic ALS, they are not found in some sporadic ALS cases as well as cases of familial ALS caused by SOD1 mutations.^26,27^ In a previous study, CysC was immunostained in remaining neurons as a condensed form and activated astrocytes in SOD1^G93A^ mouse spinal cord.^24^ Therefore, we first confirmed the localization of endogenous murine CysC and its alternations in SOD1^G93A^ mouse spinal cord (Figure 1). The punctate staining of murine CysC was observed in SOD1 wild-type mouse spinal cord. Murine CysC immunoreactivity was strongly positive in the anterior horn neurons, whereas it was weak or negative in the posterior horn neurons and the glial cells (Figure 1a). Murine CysC was partially co-localized with lysosome-associated membrane protein 2 (LAMP-2), a marker protein of lysosomes (Figure 1b). In SOD1^G93A^ mouse spinal cord, murine CysC immunoreactivity was generally reduced and condensed in the remaining neurons, and it was also found in the outside of neurons (Figures 1c and d). Interestingly, murine CysC was still immunostained in remaining, normal-appearing neurons (Figure 1c, arrows), whereas murine CysC immunoreactivity was almost undetectable in shrunken neurons (Figure 1c, arrowheads). These results are similar to that of human sporadic ALS cases,^13^ suggesting that murine CysC immunoreactivity correlates with motor neuronal survival. Moreover, LAMP-2 immunoreactivity was also diminished in the SOD1^G93A^ anterior horn neurons (Figure 1d), implying the involvement of lysosomal dysfunction in motor neuronal degeneration.

### CysC protects N2a cells against mutant SOD1-mediated neurotoxicity

**Figure 2:**
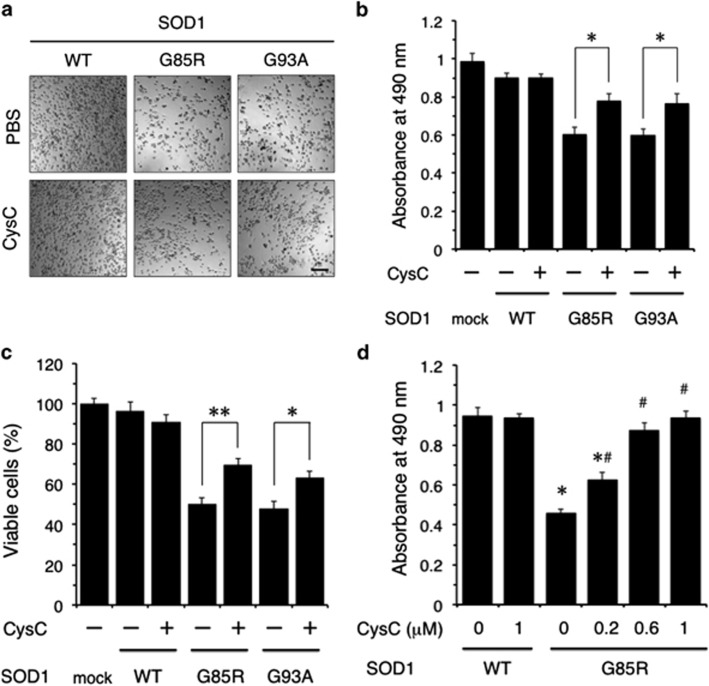
CysC protects N2a cells against mutant SOD1-mediated cytotoxicity. (**a**–**c**) N2a cells were transfected with SOD1^WT^ (WT), SOD1^G85R^ (G85R) or SOD1^G93A^ (G93A), and incubated for 48 h in the differentiation medium with or without 0.2 *μ*M CysC. Light microscopy images (**a**), the cell viability measured by the MTS assay (**b**), or quantification of viable cells with trypan blue staining (**c**) were shown. (**d**) Dose-dependent cytoprotection of CysC. Transfected N2a cells were incubated with CysC at the indicated concentrations. Cytoprotective activity of CysC was measured by the MTS assay. All data are expressed as means±standard error of the mean (S.E.M.) from three independent experiments, each performed in triplicate. *P<0.05, **P<0.01 in (**b**) and (**c**). *P<0.05 compared to SOD1^WT^ without CysC, ^#^*P*<0.05 compared to SOD1^G85R^ without CysC in D. Scale bar: 200 *μ*m

In order to investigate whether CysC is involved in motor neuronal survival, we examined the neuroprotective activity of exogenously added recombinant human CysC against mutant SOD1-mediated toxicity. Phase microscopic images showed that both of G85R and G93A mutant SOD1 were toxic to differentiated N2a cells and the addition of CysC to the culture medium reduced their toxicity (Figure 2a). The neuroprotective effect of CysC against mutant SOD1-mediated toxicity was further confirmed by the 3-(4,5-dimethylthiazol-2-yl)-5-(3-carboxymethoxyphenyl)-2-(4-sulfophenyl)-2H-tetrazolium (MTS) assay (Figure 2b) and by quantitation of the live cells excluding trypan blue dye (Figure 2c). To evaluate the concentration dependence of neuroprotection by CysC, we added CysC into the culture medium at dose of 0, 0.2, 0.6 and 1 *μ*M. As shown in Figure 2d, CysC protected the N2a cells in a concentration-dependent manner. These results provide strong evidence that CysC is involved in neuroprotection against mutant SOD1-mediated toxicity.

### CysC reduces toxic SOD1 species by induction of autophagy

**Figure 3:**
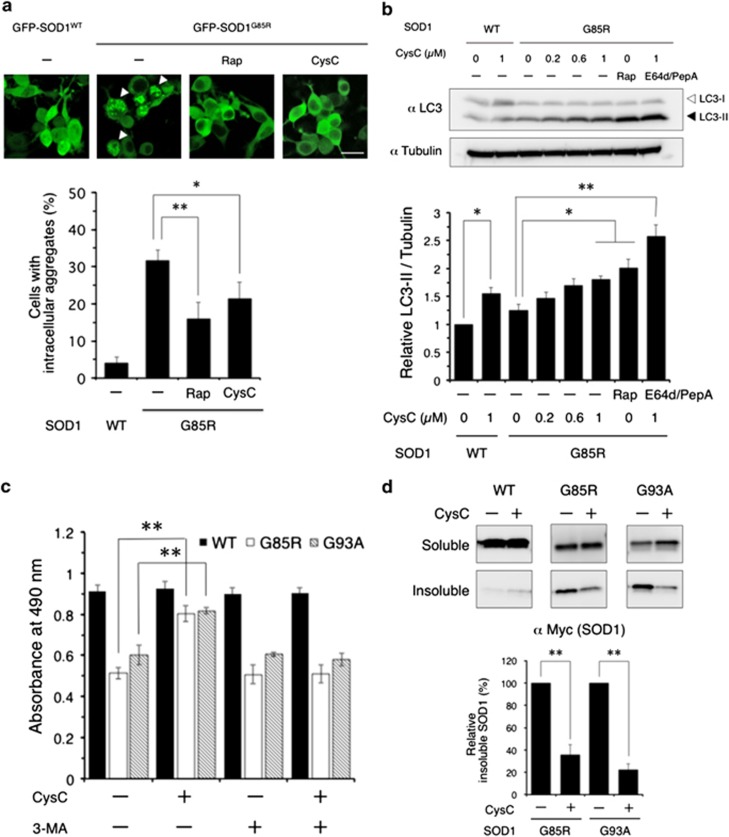
CysC induces autophagy and reduces detergent-insoluble mutant SOD1 proteins in cells. (**a**) Representative fluorescent microscopy images of N2a cells expressing GFP-SOD1 incubated for 24 h with rapamycin (Rap.; 300 nM) or CysC (0.2 *μ*M). The arrowheads indicate the intracellular mutant SOD1 aggregates. Quantified data are expressed as means±S.E.M. from three independent experiments. In each experiment, at least 100 cells were counted. (**b**) Induction of LC3-II, an autophagy marker, by CysC. N2a cells were transfected with SOD1^WT^ or SOD1^G85R^, and incubated with CysC at the indicated concentrations for 12 h. Lysates were analyzed by immunoblotting with anti-LC3 and *α*-tubulin antibodies. Relative levels of LC3-II normalized by the expression of *α*-tubulin were quantified. (**c**) The effect of autophagy inhibition on neuroprotection by CysC. N2a cells expressing wild-type or mutant SOD1 were treated with CysC (0.2 *μ*M) and/or 3-MA (10 mM). Cell viability was measured by the MTS assay. Data are expressed as means±S.E.M. from three independent experiments. Each experiment was performed in triplicate. (**d**) Reduction of Triton-insoluble mutant SOD1 species by CysC. After 24 h of CysC treatment, N2a cells expressing SOD1 constructs were lysed with 1% Triton X-100. Triton-insoluble fractions were resuspended with 2% sodium dodecyl sulfate and analyzed by immunoblotting. **P<0.01. *P<0.05. Scale bar: 50 *μ*m

A previous study demonstrated that CysC protected neuronal cells against various stresses by induction of autophagy.^16^ To examine whether autophagy is involved in the neuroprotective activity of CysC against SOD1-mediated toxicity, we first evaluated the number of intracellular SOD1 aggregates with fluorescent microscopy. As shown in Figure 3a, the green fluorescent protein (GFP)-fused SOD1^G85R^ mutant formed intracellular aggregates in about 30% of total transfected N2a cells. Rapamycin, a representative inducer of autophagy, remarkably reduced these intracellular aggregates of SOD1^G85R^ to about 50% of untreated cells. CysC treatment also decreased the aggregates like rapamycin did to about 70% of untreated cells, although difference in the degree of reducing aggregates between CysC and rapamycin treatment was not statistically significant. These data imply that CysC protected N2a cells through induction of autophagy. LC3, a selective marker of autophagy, normally appears as a cytosolic form (LC3-I) and becomes cleaved, membrane-bound form (LC3-II) under autophagosome formation process. Western blot analyses showed that CysC enhanced the formation of LC3-II (Figure 3b). Lysosomal proteinase inhibitors, E64d and pepstatin A, further increased the level of LC3-II, indicating the upregulation of autophagy by CysC. Interestingly, the induction of autophagy was independent of the transfected SOD1 species, suggesting that the regulation of autophagy by CysC is physiologically constitutive and independent of SOD1-mediated toxicity (Figure 3b). Next, in order to confirm the relationship between neuroprotective activity of CysC against mutant SOD1-mediated toxicity and induction of autophagy, the MTS assay was performed in the presence of 3-methyladenine (3-MA), an inhibitor of macroautophagy. As shown in Figure 3c, the protective effects of CysC were completely abolished by 3-MA treatment. Moreover, CysC treatment remarkably reduced Triton X-100-insoluble SOD1 mutants (Figure 3d), suggesting degradation of the misfolded toxic SOD1 species by autophagy. All of these data indicate that the induction of autophagy is essential for the neuroprotective activity of CysC.

### CysC induces autophagy via AMPK-mTOR pathway

**Figure 4:**
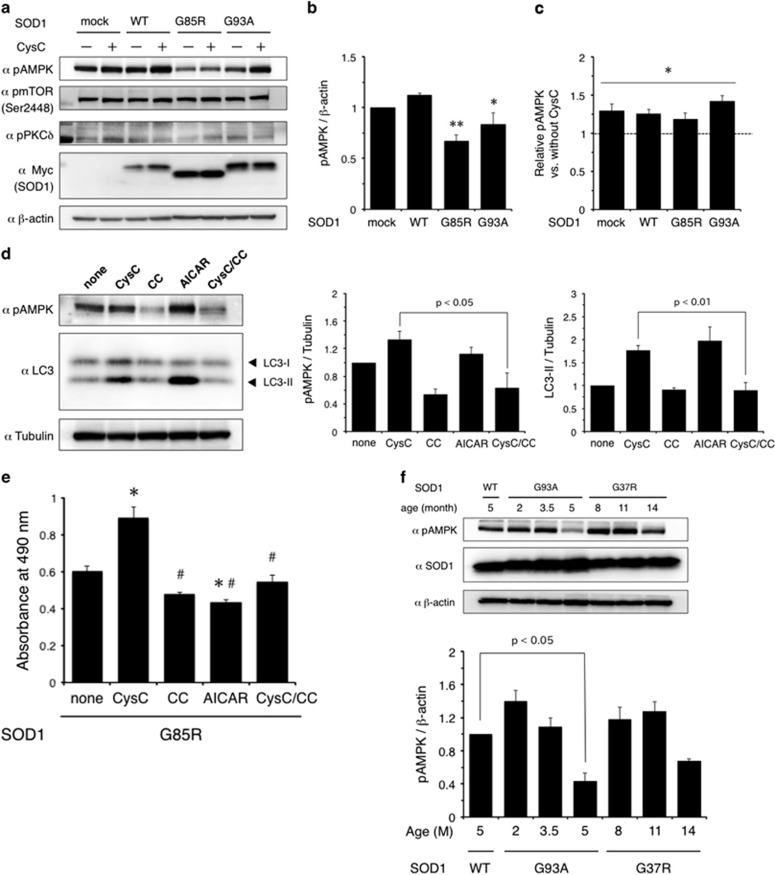
CysC regulates AMPK activity during the induction of autophagy. (**a**) Immunoblotting analysis of autophagy regulators. N2a cells expressing SOD1 were treated with or without CysC (1 *μ*M) for 6 h. The lysates were analyzed by immunoblotting using antibodies for phosphorylated AMPK (pAMPK), phosphorylated mTOR (pmTOR), phosphorylated PKC*δ* (pPKC*δ*), Myc and *β*-actin. (**b**) Inactivation of AMPK by mutant SOD1 expression. Each relative pAMPK level normalized by *β*-actin in (**a**) is quantified. *P<0.05, **P<0.01 *versus* mock. (**c**) Activation of AMPK by CysC treatment. Relative levels of pAMPK for CysC-treated samples normalized by that of PBS-treated control, which is shown as the broken line, in (**a**) were quantified. *P<0.05 *versus* PBS-treated controls. (**d**) CysC induced autophagy through the AMPK activation. N2a cells were treated with CysC (1 *μ*M), CC (5 *μ*M) or AICA-riboside (AICAR, 5 mM) for 12 h. The lysates were analyzed by immunoblotting using antibodies against pAMPK, LC3 and Tubulin (left panel). Quantification of immunoblots was plotted (right panel). (**e**) The effect of pAMPK activation on neuroprotection by CysC. N2a cells expressing G85R SOD1 mutant were treated with CysC (0.2 *μ*M), CC (5 *μ*M) or AICAR (5 mM). Cell viability was measured by the MTS assay. Data are expressed as means±S.E.M. from three independent experiments. Each experiment was performed in triplicate. *P<0.01 compared to non-treated control, ^#^*P*<0.01 compared to CysC-treated one. (**f**) Immunoblotting analysis of pAMPK in SOD1 transgenic mouse spinal cords. The spinal cord lysates from the transgenic mice at indicated ages were analyzed for the levels of pAMPK, SOD1 and *β*-actin (upper panel). The expression levels of pAMPK were normalized by *β*-actin (lower panel). All data are expressed as means±S.E.M. from three independent experiments

CysC has been shown to inhibit the mammalian target of rapamycin (mTOR) complex, which suppresses autophagy.^16^ However, the mechanisms through which CysC inhibits mTOR are still uncertain. To identify the signal transduction pathway mediated by CysC, we investigated the following pathways that regulate mTOR activity. (i) AMP-activated kinase (AMPK) is a regulator of cellular metabolism and energy consumption. AMPK inactivates mTOR under malnutritic conditions, such as those involving a low glucose supply.^28^ (ii) Akt is a major signal transducer that promotes protein synthesis, cellular proliferation and cell survival. Akt phosphorylates mTOR at Ser2448 and induces its activity.^29^ (iii) Protein kinase C *δ* (PKC*δ*) is activated in the spinal cord of the wobbler mouse, an ALS model.^30^ PKC*δ* activates mTOR via the transglutaminase-Akt pathway.^31^ In order to examine the effects of CysC on these pathways, N2a cells expressing SOD1 were treated with 1 *μ*M CysC for 8 h and analyzed by immunoblotting (Figure 4a). AMPK activation was remarkably inhibited when SOD1 mutants were expressed (Figures 4a and b) and the reduced activation of AMPK by SOD1 mutants was partially recovered by CysC treatment (Figures 4a and c). In addition to this, compound C (CC), an AMPK inhibitor, clearly inhibited the AMPK activation and the conversion to LC3-II by CysC treatment (Figure 4d). These data indicate that CysC induces autophagy through the AMPK-mTOR pathway, whereas the activities of the other mTOR-regulating factors, Akt and PKC*δ*, were not affected by CysC treatment. To examine the role of AMPK activation in the neuroprotection by CysC, we measured the cell viability with CysC, AMPK inhibitor or activator (Figure 4e). CC also inhibited the neuroprotective activity of CysC. However, AICA-riboside (AICAR), an AMPK activator, exacerbated the mutant SOD1-mediated toxicity. These data indicate that AMPK activation is essential but not sufficient for the neuroprotection by CysC. To confirm the changes in AMPK activity *in vivo*, we analyzed the spinal cord lysates of SOD1 transgenic mice at various ages (Figure 4f). The phosphorylation levels of AMPK were reduced at the end stages of the disease (Figure 4f). This is consistent with the results of the N2a cells and implies that a reduction of AMPK activity is involved in the disease.

### CysC is transduced into neuronal cells via clathrin-dependent endocytotic pathway

**Figure 5:**
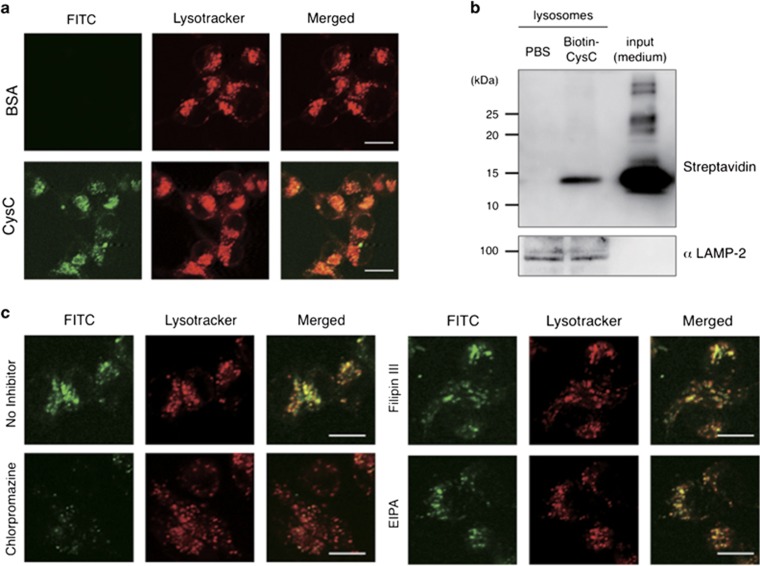
Transduction of exogenously added CysC into N2a cells. (**a**) Transduction and localization of CysC to lysosomes in N2a cells. FITC-labeled CysC or bovine serum albumin (1 *μ*M) was added to N2a cells for 3 h, and the cells were then briefly treated by LysoTracker and analyzed by confocal microscopy. (**b**) Immunoblotting detection of transduced full-length CysC in purified lysosomal fractions. Biotin-conjugated transduced CysC was detected by horseradish peroxidase-streptavidin. Immunoblot using anti-LAMP-2 antibody indicates enrichment of lysosome. (**c**) Clathrin-dependent transduction of CysC. N2a cells were pre-treated with chlorpromazine (25 *μ*M), filipin III (5 mg/ml) or 5-(N-Ethyl-N-isopropyl) amiloride (EIPA) (25 *μ*M) for 1 h. Then, the cells were incubated with FITC-labeled CysC for 1 h, and analyzed by confocal microscopy. Scale bars: 25 *μ*m

CysC transduction into some human non-neuronal cell lines^32,33^ has been previously reported. However, it has not been clarified whether CysC is also transduced into neuronal cells. Moreover, the transduction pathway by which CysC is transduced into neuronal cells is unknown. To address these questions, we added fluorescein isothiocyanate (FITC)-labeled CysC (FITC-CysC) into the culture medium. We found that CysC was transduced into neuronal cells and localized to lysosomal acidic components, which were labeled by Lysotracker-Red, as like in the case of other cells (Figure 5a). Immunoblot analyses of isolated intact lysosomes of the cells treated with biotinylated-CysC (Biotin-CysC) revealed intact full-length CysC delivery (Figure 5b). There are multiple endocytotic pathways including clathrin-dependent endocytosis, lipid raft-caveolae-dependent endocytosis and macropinocytosis. To determine the endocytotic pathway responsible for CysC transduction, we administered the pathway-specific inhibitors, chlorpromazine (clathrin-dependent endocytosis),^34^ Filipin-III (lipid raft-caveolae-dependent endocytosis)^35^ or 5-(N-Ethyl-N-isopropyl) amiloride (macropinocytosis)^36^ into the cells. Although Filipin-III and 5-(N-Ethyl-N-isopropyl) amiloride did not inhibit CysC transduction at all, chlorpromazine clearly inhibited it (Figure 5c). These data showed that CysC is transduced into the cells via the clathrin-dependent pathway and localized to lysosomes.

### Transduced CysC leaks from lysosome and forms aggregates under oxidative stress conditions

**Figure 6:**
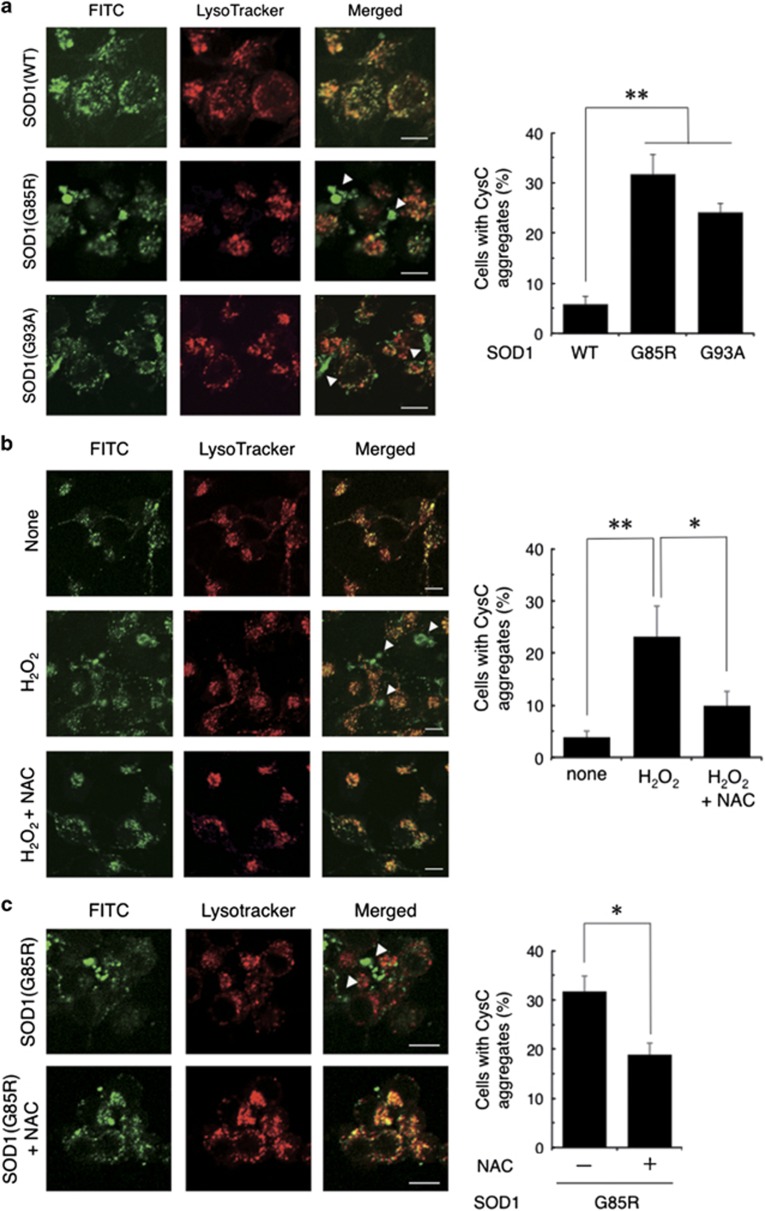
Stress-induced leakage from the lysosomes and aggregation of CysC in N2a cells. (**a**) Leakage and aggregation of CysC caused by mutant SOD1 expression. N2a cells transiently expressing SOD1 were incubated with 0.5 *μ*M FITC-CysC for 24 h, treated with LysoTracker and analyzed by confocal microscopy. (**b**) H_2_O_2_-induced aggregation of CysC and effects of N-acetyl cysteine (NAC). N2a cells were incubated for 24 h in the medium containing 0.5 *μ*M FITC-CysC and 50 *μ*M H_2_O_2_ with or without 100 *μ*M NAC. Cells were treated with LysoTracker and observed by confocal microscopy. (**c**) NAC prevented mutant SOD1-induced aggregation of CysC. N2a cells expressing G85R SOD1 mutant were incubated for 24 h in the medium containing 0.5 *μ*M FITC-CysC with or without 100 *μ*M NAC. Cells were treated with LysoTracker and observed by confocal microscopy. The arrowheads indicate the leaked and aggregated CysC in the cytosol. (**a**–**c**, right panel) Cells with CysC aggregates were quantified. **P<0.01. *P<0.05. Scale bars: 25 *μ*m

Intriguingly, transduced CysC was leaked from the lysosomes and aggregated in the cytosol when the G85R or G93A SOD1 mutant was expressed (Figure 6a), whereas wild-type SOD1 expression did not cause the CysC leakage. Previous studies have revealed that lysosomal membrane permeabilization (LMP) is induced by various stresses including oxidative stress.^37,38^ In order to investigate the possible involvement of oxidative stress in CysC leakage, we treated N2a cells with hydrogen peroxide (H_2_O_2_) and found that H_2_O_2_ treatment caused CysC leakage, which was similar to the effects of the mutant SOD1 expression (Figure 6b). Moreover, N-acetyl-L-cysteine, a scavenger of reactive oxygen species, inhibited H_2_O_2_-induced CysC release from the lysosomes. Furthermore, N-acetyl-L-cysteine also inhibited the leakage of CysC from lysosomes when G85R SOD1 mutant was expressed (Figure 6c). These data suggest that LMP caused by oxidative stress is a major cause of CysC leakage from lysosomes.

### CatB inhibitory activity of CysC is required for its neuroprotection

**Figure 7:**
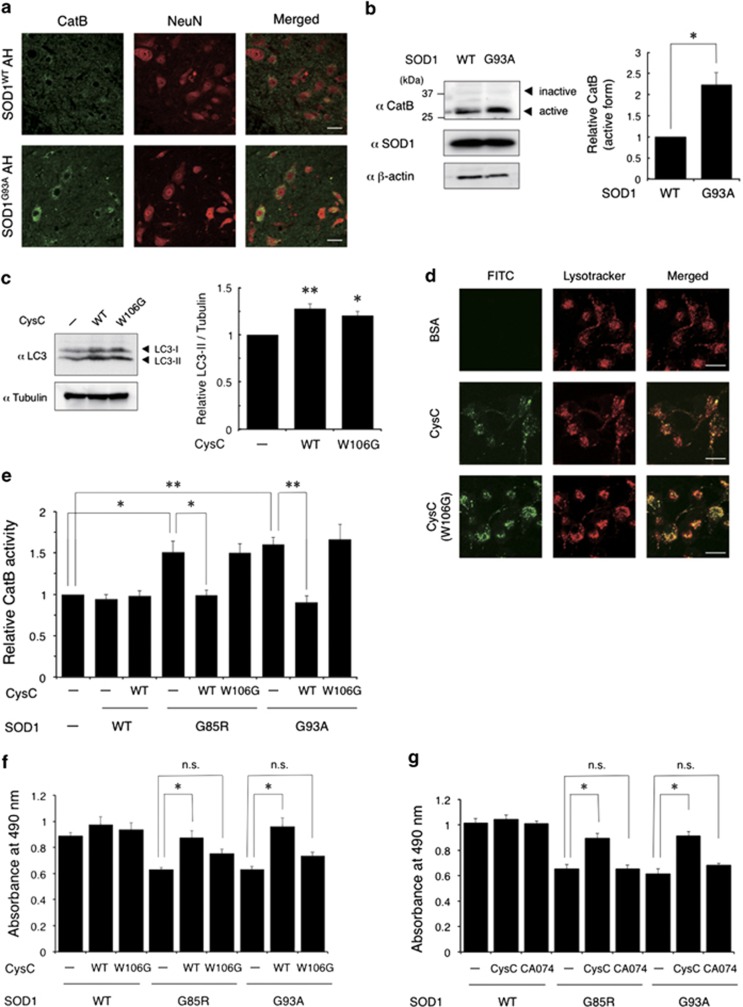
Transduced CysC exerts neuroprotective activity through the inhibition of Cathepsin B. (**a**) Expression of CatB in mouse spinal cords. Transverse sections in anterior horn (AH) of SOD1^WT^ or SOD1^G93A^ 5-month-old mouse spinal cords were analyzed by confocal microscopy. Scale bar: 50 *μ*m (**b**) Induction of active form CatB in the SOD1^G93A^ mouse spinal cord. Lysates of SOD1^WT^ or SOD1^G93A^ 5-month-old mouse spinal cords were analyzed by immunoblotting. Quantification of CatB relative to *β*-actin in immunoblots (bottom). (**c**) Induction of autophagy by wild-type (WT) or W106G CysC. N2a cells were treated with CysC (0.6 *μ*M) for 24 h. The lysates of the cells were analyzed by immunoblotting. (**d**) Intracellular transduction of WT and W106G CysC. N2a cells were treated with FITC-CysC (1 *μ*M) for 3 h and observed by confocal microscopy. Scale bar: 25 *μ*m (**e**) Enzymatic activities of CatB were measured by the luciferase assay with a CatB-specific substrate. N2a cells transiently expressing mutant SOD1 species were treated with CysC (1 *μ*M) for 24 h. The lysates of the cells were used for measuring CatB activity. (**f** and **g**) Protective effects of WT and W106G CysC (**f**), and a CatB-specific inhibitor CA-074 (**g**). The viability of N2a cells was examined by the MTS assay after incubation for 48 h in the differentiation medium with WT or W106G CysC (0.2 *μ*M), or CA-074 methyl ester (10 *μ*M). These experiments were independently performed three times with triplicate samples. All data were shown as means±S.E.M. **P<0.01. *P<0.05

It is of interest whether the CatB inhibitory activity of CysC is also involved in its neuroprotective activity, because the previous reports have shown that CatB is activated in ALS pathology^23,24^ and involved in motor neuronal degeneration.^25^ First, we confirmed the activation of CatB in the mutant SOD1 model. The immunoreactivity of CatB and the amount of the CatB active form was substantially increased in SOD1^G93A^ mouse spinal cord, which was similar to the findings of previous studies (Figures 7a and b). Next, to examine the inhibitory activity of CysC treatment on CatB activation by mutant SOD1 expression, we created a W106G CysC mutant that specifically lacked the inhibitory activity against CatB.^39^ Similar to the wild-type, the W106G mutant induced autophagy (Figure 7c) and transduced into N2a cells (Figure 7d). As shown in Figure 7e, the CatB activity induced by mutant SOD1 was completely inhibited by wild-type CysC treatment, whereas the W106G CysC mutant did not inhibit the activation of CatB, suggesting that transduced CysC functioned as the endogenous intracellular CatB inhibitor. Surprisingly, CysC treatment did not affect the CatB activity at all in the absence of mutant SOD1, implying that CysC was effective only to inhibit the aberrant activation of CatB. Moreover, to assess the contribution of CatB inhibition by CysC to its neuroprotective activity, we examined the neuroprotective activity of the wild-type or W106G CysC mutant to mutant SOD1-mediated toxicity by the MTS assay. As shown in Figure 7f, W106G CysC did not reduce the mutant SOD1-mediated toxicity at all. This suggests that CatB inhibitory activity was also essential for the neuroprotective activity of CysC as well as the induction of autophagy. Moreover, a CatB-specific inhibitor CA-074 did not improve the viability of the N2a cells (Figure 7g), indicating that the CatB inhibitory activity was essential but not sufficient to protect neurons as well as the induction of autophagy.

### CysC protects primary cultured motor neurons from mutant SOD1-mediated toxicity

**Figure 8:**
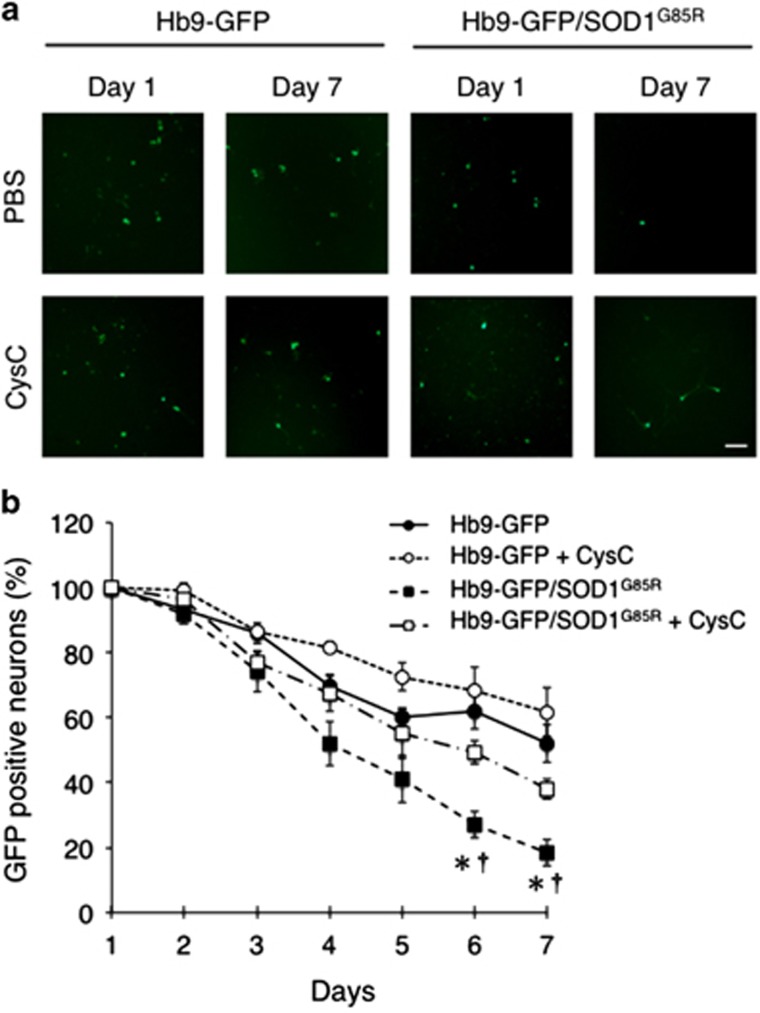
CysC protects primary cultured motor neurons *in vitro*. (**a**) Representative fluorescent images of the primary spinal cord mix culture from Hb9-GFP or Hb9-GFP/SOD1^G85R^ transgenic mouse embryos. The spinal mix culture was treated with or without CysC. GFP-positive cells represent motor neurons. Scale bar: 100 *μ*m. (**b**) The viability of motor neurons in the spinal cord mix culture of Hb9-GFP (closed circle, solid line), Hb9-GFP with CysC (open circle, dotted line), Hb9-GFP/SOD1^G85R^ (closed square, broken line) and Hb9-GFP/SOD1^G85R^ with CysC (open square, dashed line). GFP-positive motor neurons were quantified. Data are expressed as means±S.E.M. from three independent experiments. The cells were counted in nine random fields in each experiment (*n*=3). *P<0.05 compared to Hb9-GFP without CysC. †*P<*0.01 compared to Hb9-GFP/SOD1^G85R^ with CysC

In order to confirm the protective effect of CysC on primary motor neurons, we examined motor neuronal survival in a primary neuron-glia mix culture derived from Hb9-GFP/SOD1^G85R^ mouse embryos. As Hb9-GFP mice have been used to identify motor neurons in mice,^40^ the number of GFP-positive cells represents surviving motor neurons (Figure 8a). Mixed culture derived from SOD1^G85R^ embryos showed an accelerated decrease of motor neurons compared with that of the wild-type one and CysC treatment markedly improved the viability of SOD1^G85R^ motor neurons (Figure 8b). This result indicates that CysC exerts a neuroprotective activity against mutant SOD1-mediated toxicity on primary cultured motor neurons as well as N2a cells.

## Discussion

In the present study, we demonstrated the neuroprotective activity of CysC against mutant SOD1-mediated toxicity. CysC protected neuronal cells through two distinct pathways: (i) induction of autophagy through AMPK activation and (ii) inhibition of aberrantly activated CatB. Furthermore, the coordinated activation of these two pathways was required for neuroprotection by CysC.

Autophagy is a major degradation pathway of misfolded or unfolded proteins as well as the ubiquitin-proteasomal pathway^41^ and regulation of basal autophagy is crucial for neural survivals.^42,43^ Impairment of the ubiquitin-proteasomal pathway, which has been reported in both SOD1-related^17,18^ and SOD1-unrelated^19,20^ ALS models implies that activation of the autophagy pathway may complementally contribute to degradation of abnormal toxic proteins. Indeed, inducers of autophagy such as progesterone, trehalose or rapamycin showed neuroprotective effects by degrading toxic species.^21,22,29,44^ Our finding that CysC induces autophagy to protect neurons is consistent with these previous results. Reduction of the intracellular aggregates (Figure 3a) and the Triton-insoluble mutant SOD1 protein (Figure 3d) suggested induction of autophagic protein degradation by CysC. Furthermore, 3-MA treatment demonstrated that induction of autophagy is essential for the neuroprotective activity of CysC (Figure 3c). As induction of autophagy by CysC also protects neurons against various stresses other than SOD1-mediated toxicity^16^ and rapamycin protected neurons in mice expressing TAR DNA binding protein 43 (TDP-43), another disease-linked protein accumulated in sporadic ALS and frontotemporal lobar degeneration,^22^ CysC treatment may also be useful for SOD1-unrelated ALS as well as the SOD1-linked one. Although autophagy is generally considered as a neuroprotective pathway, autophagy inducers are not effective^45^ or can even exacerbate the disease progression.^46^ We demonstrated that induction of autophagy is essential for neuroprotection by CysC (Figure 3c). However, induction of autophagy by AICAR treatment (Figures 4d and e) or W106G CysC mutant (Figures 7c and f) alone was insufficient to protect neuronal cells against the mutant SOD1-mediated toxicity. These data suggest that activation of multiple neuroprotective pathways including autophagy is required for neuroprotection. In addition to this, it has recently been suggested that these inconsistent results might be due to the side effects of rapamycin, which are independent of autophagy induction.^47^ In light of this, CysC-mediated autophagy is one of the neuroprotective mechanisms against mutant SOD1-mediated neurotoxicity and may be a promising candidate for neuroprotection.

We demonstrated that CysC activated AMPK to inhibit mTOR (Figure 4). AMPK was inactivated both in the *in vitro* and *in vivo* models of SOD1-linked ALS, suggesting that metabolic aberration is involved in the disease and that CysC possibly contributes to restore intracellular metabolic homeostasis. Moreover, inducing autophagy by CysC through AMPK activation even without mutant SOD1 implies the idea that CysC is a regulator of basal autophagy required for neuronal survival. However, in a previous study, the reduced activity of AMPK improved the motor activity of neurons in *C. elegans* without halting neurodegeneration.^48^ Their findings seem to be in contradiction to our findings. One possible interpretation is that coordinated activation of AMPK is required to inhibit neurodegeneration. As we demonstrated, AMPK inhibition by CC prevented the neuroprotection by CysC (Figure 4e), indicating AMPK activation is essential. On the other hand, we also showed that AMPK-specific activation by AICAR exacerbated the mutant SOD1-mediated toxicity (Figure 4e), which is consistent with a previous study,^48^ and CatB inhibitory activity of CysC is also required for neuroprotection (Figure 7). These data indicate that AMPK-specific activation is not sufficient to protect the neuronal cells and, eventually, might be toxic. Therefore, we suggest that the synergetic regulation of intracellular signaling pathways should be required for neuroprotection.

Aberrant CatB activation is correlated with motor neuronal death in sporadic ALS cases^23^ and the SOD1^G93A^ mice model.^24^ We showed that inhibition of CatB activity by CysC was also essential for its neuroprotective activity (Figure 7), supporting the idea that aberrant proteolysis mediated by CatB is highly toxic to motor neurons. It should be noted that the W106G CysC mutant did not rescue the cells at all (Figure 7f), regardless of its ability for intracellular transduction and autophagy induction. Moreover, CatB-specific inhibition by CA-074 was also not protective (Figure 7g). These results suggest that autophagy is less protective alone and synergistic activation of multiple neuroprotective pathways is crucial to ameliorate neurodegeneration in ALS.

Interestingly, transduced CysC inhibited CatB only when CatB was activated by stress without affecting basal CatB activity, suggesting that the transduced CysC was inactivated in the lysosomes and reactivated under stress conditions. As CysC forms a reversible dimer and/or oligomer under low pH conditions *in vitro*^49^ and dimeric CysC does not inhibit CatB,^50^ acidic condition in the lysosomes might contribute to this stress-inducible response of CysC. CysC was leaked into cytosol only when mutant SOD1 species were expressed (Figure 6), supporting this notion. Moreover, involvement of lysosomal dysfunction in neurodegeneration, especially LMP and LMP-caused lysosomal proteinase leakage possibly including CatB, is also implicated by our data (Figures 1d and 6) and previous studies.^37,38^ Therefore, CysC released from lysosome by LMP may specifically inhibit aberrant cytosolic proteolysis caused by leaked CatB.

CysC was transduced into N2a cells and localized to lysosomes, which was like that seen in the other cell lines^32,33^ through clathrin-dependent endocytosis (Figure 5). We also revealed that transduced CysC leaked into the cytosol from the lysosomes and aggregated oxidative stress-dependently (Figures 6b and c). These data suggest that stress-induced CysC release from the lysosomes is a first step in forming Bunina bodies. However, Bunina bodies are not found in familial ALS patients with SOD1 mutations.^26,27^ One possible interpretation is that the amount of CysC in neurons is not enough to form Bunina bodies in those SOD1-linked ALS cases. Indeed, the immunoreactivity of CysC in SOD1^G93A^ mouse spinal cord was apparently reduced (Figure 1). In addition to this, increased CysC immunoreactivity in non-neuronal cells (Figure 1c) implies dysfunction of the CysC secretory pathway. Therefore, the downregulation of CysC level in the neurons carrying SOD1 mutations may inhibit the formation of Bunina bodies. Further studies are required to investigate the mechanism of Bunina bodies' formation.

We demonstrate here that CysC, a main component of Bunina bodies in ALS, is an endogenous neuroprotective factor that acts through coordinated activation of two distinct neuroprotective pathways: induction of autophagy and inhibition of aberrant CatB activity. We expect further investigations of the mechanisms through which CysC accumulates and maintains proteostasis in motor neurons should clarify the role of Bunina bodies in ALS. Furthermore, targeting CysC in motor neurons may become a novel therapeutic strategy for ALS.

## Materials and Methods

### Antibodies

Antibodies against phosphorylated PKC*δ* (Thr507), AMPK*α* 1/2 (Thr172) and anti- LAMP-2 antibody were obtained from Santa Cruz Biotechnology Inc. (Santa Cruz, CA, USA). The anti-phosphorylated mammalian target of rapamycin (mTOR) (Ser2448) antibody was obtained from Cell Signaling Technology Inc. (Danvers, MA, USA). Antibodies against NeuN, CatB and CysC were obtained from EMD Millipore Corp. (Billerica, MA, USA). Alexa Fluor-conjugated secondary antibodies were purchased from Life Technologies Corp. (Grand Island, NY, USA). We also used the following commercially available antibodies: anti-c-myc (Roche, Basel, Switzerland), anti-tubulin, anti-*β*-actin (both from Sigma-Aldrich Co. LLC St. Louis, MO, USA) and anti-LC3 (Novus Biologicals LLC, Littleton, CO, USA). Rabbit anti-human SOD1 was raised in our laboratory against a recombinant human SOD1 peptide (24–36) and purified with protein A.^51^

### Transgenic mice

Wild-type, mutant SOD1 (B6.Cg-Tg(SOD1-G37R) 1Dwc/J), (B6.Cg-Tg(SOD1-G85R) 148Dwc/J), (B6.Cg-Tg(SOD1-G93A) 1Gur/J) and Hb9-GFP (B6.Cg-Tg(Hlxb9-GFP) 1Tmj/J) transgenic mice were obtained from the Jackson Laboratory (Bar Harbor, ME, USA) or were gifts from Dr. Don Cleveland (University of California, San Diego). The mice were genotyped by polymerase chain reactions with the following sense and antisense primers: 5′-CATCAGCCCTAATCCATCTGA-3′, 5′-CGCGACTAACAATCAAAGTGA-3′, respectively. The mice were housed and treated in compliance with the requirements of the Animal Care and Use Committee of RIKEN Brain Science Institute and Nagoya University.

### Immunofluorescence staining

Immunofluorescence staining was performed as described previously.^52^ In brief, after blocking, the sections were incubated with anti-CysC (1 : 100), anti-Cathepsin B, anti-NeuN (1 : 500) and/or anti-LAMP-2 (1 : 100) overnight at 4 °C. Bound antibodies were detected with Alexa Fluor 488-conjugated anti-rabbit IgG, Alexa Fluor 594-conjugated anti-mouse IgG and Alexa Fluor 650-conjugated anti-rat IgG antibodies (all 1 : 1000). Immunostained images were obtained by confocal laser scanning microscopy (LSM 5 Exciter, LSM-700; Carl Zeiss AG, Oberkochen, Germany) and the equipped software (Zen; Carl Zeiss AG).

### Construction, expression and purification of recombinant human CysC protein

The DNA fragment containing human CysC (lacking the 26 amino acids leader sequence of the precursor CysC) was amplified by polymerase chain reactions and cloned into the pET-20b(+) vector (EMD Millipore) under *pelB* signal sequence for potential periplasmic localization. Overexpression of CysC was induced in *Escherichia coli* strain Rosetta (DE3) (EMD Millipore) by treatment with 0.3 mM isopropyl-*ß*-D-thiogalactopylanoside for 4.5 h at 30 °C and CysC protein was purified according to the osmotic shock protocol (EMD Millipore) to prepare for periplasmic fraction with some modifications.^53,54^ In brief, the cells were resuspended in an equal volume of 30 mM Tris-HCl (pH 9.0), 20% (w/w) sucrose. Then, ethylenediaminetetraacetic acid was added to 1 mM. The cells were agitated for 10 min at room temperature and centrifuged at 13 000 × *g* for 10 min. The pellet was resuspended in an equal volume of ice-cold 10 mM Tris-HCl (pH 9.0) and 5 mM MgSO_4_, then it was agitated for 10 min at 4 °C. The cell debris was removed by centrifugation at 13 000 × *g* for 10 min at 4 °C and the supernatant was loaded onto a DEAE sepharose anion-exchange column equilibrated with 50 mM Tris-HCl (pH 9.0). The flow-through was collected and concentrated. Purified CysC was dialyzed against 10 mM Tris-HCl (pH 9.0) and 150 mM NaCl. Endotoxin was removed from the protein solutions by phase separation using Triton-X 114 (Wako Pure Chemical Industries Ltd., Osaka, Japan).^55,56^ The amounts of Triton X-114 were removed by ultrafiltration with an Amicon Ultra centrifugal unit (EMD Millipore). The protein concentration of CysC was determined spectrophotometrically with an extinction coefficient of 11.5 mM/cm at 280 nm.^57^

### Biotin or FITC labeling of CysC proteins

CysC was conjugated to D-biotin, succinimidyl azide (Life Technologies) or FITC (Dojindo Laboratories, Kumamoto, Japan) according to the manufacturers' instructions. Labeled CysC was purified with G25 gel chromatography to eliminate any unconjugated reagents. The conjugation of biotin to CysC was confirmed by an immunoblotting assay using horseradish peroxidase conjugated streptavidin (Thermo Fisher Scientific Inc., Waltham, MA, USA). The concentrations of CysC protein and FITC dye were calculated on the basis of their absorbance at 280 and 494 nm, respectively. The molar ratio of dye per protein was determined to be 0.9–1.8.

### Cell culture

N2a cells were maintained in Dulbecco's modified Eagle's medium (DMEM) containing 4.5 g/l glucose supplemented with 10% (v/v) fetal bovine serum (FBS), 100 U/ml penicillin and 100 *μ*g/ml streptomycin (all from Life Technologies) in a humidified atmosphere containing 5% CO_2_ at 37 °C. To differentiate the cells, the cells were cultured with a differentiation medium (DMEM containing 4.5 g/l glucose supplemented with 2% (v/v) FBS and 2 mM N,N-dibutyladenosine 3′,5′-phosphoric acid (dbcAMP; Nacalai Tesque Inc., Kyoto, Japan)).

### Isolation of intact lysosomes from Neuro2a cells

Lysosomes were isolated by an Optiprep (Axis-Shield plc, Dundee, UK) gradient centrifugation as previously reported.^58,59^ Briefly, the cells treated with 1 *μ*M Biotin-CysC for 24 h were homogenized in 10 mM HEPES-KOH (pH 7.4), 0.25 M sucrose and 1 mM ethylenediamineteraacetic acid with a Potter-Elehjem tissue grinder (Wheaton, Millville, NJ, USA). The cell debris and nuclei were removed by centrifugation at 600 × *g* for 5 min at 4 °C. The post-nuclear fraction was further centrifuged at 3000 × *g* for 10 min at 4 °C to remove the mitochondrial fraction. The resultant supernatant was loaded onto an Optiprep gradient (1 ml each of 10, 12, 14, 16 and 18% (w/v)) and centrifuged at 145 000 × *g* for 2 h at 4 °C. One milliliter from the top was collected and analyzed by immunoblotting.

### Immunoblotting

N2a cells seeded at 2.0 × 10^5^ cells/ml in 6-well plates were transfected with pcDNA3.3-SOD1 expression vectors by Lipofectamine 2000 (Life Technologies). After 24 h of incubation, the medium was replaced with the differentiation medium with CysC, rapamycin (EMD Millipore, 300 nM) or E64d/Pepstatin A (both from Sigma, 10 mg/ml each). After the indicated time of incubation at 37 °C, the cells were washed with ice-cold phosphate-buffered saline (PBS) twice and harvested in TNE lysis buffer (50 mM Tris-HCl (pH 7.4), 150 mM NaCl, 1 mM ethylenediamineteraacetic acid, 1% Triton-X 100, protease inhibitor cocktail and PhosSTOP (both from Roche)). The cells or spinal cord lysates of the transgenic mice in TNE lysis buffer were sonicated and centrifuged at 15 000 × *g* for 5 min at 4 °C. The protein concentrations in the supernatants were measured by micro BCA assay kit (Thermo Fisher Scientific Inc.). Each 15 *μ*g/lane of total proteins was analyzed by immunoblotting. Densitometric analysis was performed after the chemiluminescence detection by using an image analyzer LAS-4000mini (Fuji film, Tokyo, Japan) with the equipped software (Multi Gauge: Fuji film).

### Neurotoxicity assays

Neurotoxicity was analyzed as previously reported^60^ with slight modifications. N2a cells were seeded at 5.0 × 10^4^ cells/ml in poly-D-lysine coated 96-well plates in DMEM containing 1.0 g/l glucose supplemented with 10% (v/v) FBS. After transfection, the cells were differentiated for 48 h in DMEM containing 1.0 g/l glucose supplemented with 2% (v/v) FBS and 2 mM dbcAMP with or without CysC, 3-methyladenine (3-MA; Sigma), CC (EMD Millipore), AICA-riboside (AICAR; EMD Millipore) or CA-074 methyl ester (EMD Millipore). The number of live cells was manually counted by trypan blue staining or estimated by CellTiter 96 AQueous One Solution Cell Proliferation Assay kit (Promega Biosciences, San Louis Obispo, CA, USA) containing MTS as described by the manufacturer. A primary glia-neuron mix culture was prepared from E12.5 embryonic spinal cord of Hb9-GFP/SOD1^G85R^ mouse. The cells were maintained in DMEM supplemented with 10% (v/v) FBS and the medium was changed every 3 days. The numbers of GFP-positive motor neuronal cells were counted manually with fluorescent microscopy.

### Examination of CysC transduction into cells with fluorescence microscopy

N2a cells were seeded at 1.0 × 10^5^ cells/ml in poly-D-lysine-coated 35 mm glass-bottomed dishes (MatTek Corp., Ashford, MA, USA). The medium was replaced with the differentiation medium containing 1 *μ*M FITC-CysC or 300 nM rapamycin at 6 h after the transfection of pcDNA3.3-SOD1 or pAcGFP-N1-SOD1. The cells were further incubated for 24 h. To determine the CysC endocytotic pathway, the cells were treated with 25 *μ*M chlorpromazine (Wako), 5 mg/ml filipin III (Enzo Life Sciences Inc., Farmingdale, NY, USA) or 25 *μ*M 5-(N-Ethyl-N-isopropyl) amiloride (Enzo) for 1 h. The medium was replaced with the differentiation one with or without 1 *μ*M FITC-CysC and incubated for another 1 h. The cells were stained with Lysotracker-Red (Life Technologies) according to the manufacturer's instructions to visualize the lysosomal acidic components. The cells were washed with PBS twice and observed by confocal laser scanning microscopy.

### CatB activity assay

N2a cells were seeded at 2.0 × 10^5^ cells/ml in 60 mm dishes and transfected with pcDNA3.3-SOD1 expression vectors by Lipofectamine 2000. After 24 h of transfection, the medium was replaced with differentiation medium containing 1 *μ*M wild-type or W106G CysC. The cells were incubated for 24 h at 37 °C in 5% CO_2_ and harvested in ice-cold PBS. Intracellular CatB activities were measured with a Cathepsin B Activity Assay kit (BioVision Inc., Mountain View, CA, USA) as described in the manufacturer's protocol.

### Statistics

Neuroprotective activity of CysC on primary cultured motor neurons was analyzed by a two-way ANOVA with subsequent post hoc *t*-tests. All other quantified data were analyzed by a one-way ANOVA with subsequent post hoc *t*-tests or by a Student's *t*-test.

## Abbreviations

AICAR: AICA-riboside
ALS: amyotrophic lateral sclerosis
AMPK: AMP-activated protein kinase
CatB: cathepsin B
CC: Compound C
CysC: cystatin C
dbcAMP: N,N-dibutyladenosine 3',5'-phosphoric acid
DMEM: Dulbecco's modified Eagle's medium
FBS: fetal bovine serum
FITC: fluorescein isothiocyanate
GFP: green fluorescent protein
H_2_O_2_: hydrogen peroxide
LMP: lysosomal membrane permeabilization
3-MA: 3-methyl adenine
mTOR: mammalian target of rapamycin
MTS: 3-(4,5-dimethylthiazol-2-yl)-5-(3-carboxymethoxyphenyl)-2-(4-sulfophenyl)-2H-tetrazolium, inner salt
N2a: neuro2a
pAMPK: phosphorylated-AMPK
PBS: phosphate buffered saline
PKC*δ*: protein kinase C *δ*
pPKC*δ*: phosphorylated-PKC*δ*
SOD1: copper-zinc superoxide dismutase
